# Screening of Duchenne Muscular Dystrophy (*DMD*) Mutations and Investigating Its Mutational Mechanism in Chinese Patients

**DOI:** 10.1371/journal.pone.0108038

**Published:** 2014-09-22

**Authors:** Chen Chen, Hongwei Ma, Feng Zhang, Lu Chen, Xuesha Xing, Shusen Wang, Xue Zhang, Yang Luo

**Affiliations:** 1 The Research Center for Medical Genomics, Key Laboratory of Medical Cell Biology, Chinese Ministry of Education, College of Basic Medical Science, China Medical University, Shenyang, China; 2 Department of Developing Pediatrics, Shengjing Hospital, China Medical University, Shenyang, China; 3 State Key Laboratory of Genetic Engineering and MOE Key Laboratory of Contemporary Anthropology, School of Life Sciences, Fudan University, Shanghai, China; 4 McKusick-Zhang Center for Genetic Medicine, State Key Laboratory of Medical Molecular Biology, Institute of Basic Medical Sciences, Chinese Academy of Medical Sciences and Peking Union Medical College, Beijing, China; Stem Cell Research Institute, Belgium

## Abstract

Duchenne muscular dystrophy (DMD) is a common X-linked recessive disease of muscle degeneration and death. In order to provide accurate and reliable genetic counseling and prenatal diagnosis, we screened *DMD* mutations in a cohort of 119 Chinese patients using multiplex ligation-dependent probe amplification (MLPA) and denaturing high performance liquid chromatography (DHPLC) followed by Sanger sequencing. In these unrelated DMD patients, we identified 11 patients with *DMD* small mutations (9.2%) and 81 patients with *DMD* deletions/duplications (del/dup) (68.1%), of which 64 (79.0%) were deletions, 16 (19.8%) were duplications, and one (1.2%) was both deletion and duplication. Furthermore, we analyzed the frequency of *DMD* breakpoint in the 64 deletion cases by calculating exon-deletion events of certain exon interval that revealed a novel mutation hotspot boundary. To explore why *DMD* rearrangement breakpoints were predisposed to specific regions (hotspot), we precisely characterized junction sequences of breakpoints at the nucleotide level in 21 patients with exon deleted/duplicated in *DMD* with a high-resolution SNP microarray assay. There were no exactly recurrent breakpoints and there was also no significant difference between single-exon del/dup and multiple-exon del/dup cases. The data from the current study provided a comprehensive strategy to detect *DMD* mutations for clinical practice, and identified two deletion hotspots at exon 43–55 and exon 10–23 by calculating exon-deletion events of certain exon interval. Furthermore, this is the first study to characterize *DMD* breakpoint at the nucleotide level in a Chinese population. Our observations provide better understanding of the mechanism for *DMD* gene rearrangements.

## Introduction

Duchenne muscular dystrophy (DMD; OMIM #310200) is an X-linked recessive disease that affects approximately 1 in 3500 male living births and results in muscle degeneration and death [1], [2]. Pathologically, DMD is characterized by rapidly progressive degeneration and necrosis of the proximal muscles and calf pseudo-hypertrophy. Most DMD patients show muscle weakness in early childhood, become wheelchair-dependent by 12 years old, and die of respiratory or cardiac failure in the late teens or early 20 s. DMD is caused by a mutation in the *DMD* gene, the largest known human gene, which is localized at chromosome Xq21.1, and covers ∼2.4 Mb with 79 exons [3], [4]. The protein product called dystrophin is an important cytoskeletal protein, which helps the cytoskeleton of each muscle fiber connect to the underlying basal lamina. Alteration or loss of dystrophin forces excess calcium into the cell membrane, resulting in excess water in the mitochondria; thus, the affected skeletal muscle will undergo dystrophy, mitochondrial dysfunction, and necrosis. To date, approximately 70% of DMD cases are caused by deletions/duplications (del/dup) of one or more *DMD* exons and 30% of cases have *DMD* mutations of nucleotide level. Deletions/duplications are the most common type of disease-causing mutation of the *DMD* gene. Deletion hotspots reside in both the distal and proximal region of the *DMD* gene, while duplications more frequently involve the *DMD* 5′ region [5]. To date, there is no effective therapy available for DMD patients. Therefore, it is essential to make a prenatal diagnosis and provide genetic counseling to reduce birth of such boys.

A great number of studies have been conducted to define *DMD* mutation patterns at the exon level in different populations [5]–[10]. For example, a previous Chinese study showed that the most frequently deleted regions occur between *DMD* exons 45 and 54 and between 3 and 22, and the most prevalently duplicated regions are at exons 3–11 and 21–37 [10]. *DMD* breakpoints mainly occur in introns 43–55 for deletion mutations and in introns 2 and 7 for duplication mutations [10].

Although several studies have shown the breakpoints of *DMD* at the nucleotide level in other populations [11]–[14], it is unknown where these occur in the Chinese population. Moreover, several molecular mechanisms give rise to non-recurrent gene rearrangements, including non-homologous end joining (NHEJ) [14]–[18], microhomology-mediated break-induced-replication (MMBIR) [19]–[21], replication errors secondary to replication fork stalling and template switching (FoSTeS) [22], aberrant firing of replication origins [11] and mechanisms associated with timing of replication [12], [23]. However, there is still much to be learned about the mutational mechanism(s) of *DMD* deletions/duplications.

Thus, in this study, we detected *DMD* mutations in 119 unrelated male Chinese DMD patients using an MLPA-DHPLC-sequencing technology. We then performed a high-resolution Affymetrix Genome-wide Human SNP Array 6.0, to precisely analyze the detailed sequence signatures for the breakpoint junctions of 21 *DMD* gene del/dup cases. The study will provide insightful information for studying the *DMD* mutation spectrum and for determining why *DMD* rearrangements are predisposed to develop on particular regions of the *DMD* gene.

## Materials and Methods

### Study population and ethics statement

In this study, we collected blood samples from 119 unrelated male patients clinically diagnosed with DMD, 59 corresponding potential female carriers, and eight amniotic fluid samples from Shengjing Hospital of China Medical University between May 2008 and August 2012. All patients entered in our study were clinically diagnosed by a developmental pediatrician according to pre-determined criteria: 1) a positive family history compatible with X-linked inheritance; 2) progressive symmetric muscular weakness (proximal greater than distal) often with calf hypertrophy; 3) symptoms present before the age of five; 4) wheelchair dependency before age 13; and 5) greater than 10-fold normal serum CK concentration. This study was approved by the ethical committee of China Medical University, and a written informed consent was obtained from the guardian of each subject. Genomic DNA from blood samples of 126 unaffected people was also collected by standard procedures with written informed consent.

### Multiplex ligation-dependent probe amplification (MLPA) analysis

Genomic DNA was extracted from peripheral venous blood or amniotic fluid using the Universal Genomic DNA Extraction Kit version 3.0 (TaKaRa, Dalian, China) according to the manufacturer's protocol. A commercial MLPA kit with probes of P034 and P035 was purchased from MRC Holland (Amsterdam, Netherlands) to detect *DMD* del/dup in these 119 unrelated DMD patients according to the manufacturer's recommended protocol. Briefly, 50∼500 ng of genomic DNA, in a volume of 5 µL Tris-EDTA, was denatured at 98°C for 5 min, cooled down, and then mixed with MLPA P034 or P035 probemix. The mixture was then heated to 95°C for 5 min and incubated at 60°C overnight for probe hybridization. After 16 h, ligation was performed with Ligase-65 enzyme at 54°C for 15 min and Ligase-65 enzyme was inactivated at 98°C for 5 min. Then, PCR amplification was performed with specific SALSA FAM PCR primers. After that, MLPA products and the Size Standard 600 were mixed together at a ratio of 80∶1. The mixture was separated by capillary electrophoresis and then analyzed using a Beckman CEQ-8000 genetic analytic system and Fragment Analysis software and processed using Coffalyser version 9.0 (http://www.mlpa.com/coffalyser/download.html). The relative peak ratio (RPR) of every single exon was plotted to its corresponding bar chart. After that, any candidate of single-exon deletions was further validated by further PCR amplification and DNA sequencing. Any single-exon duplication was confirmed by two additional independent experiments. Genomic DNA from blood samples of 30 unaffected people was included in the analysis as controls.

### Denaturing high performance liquid chromatography (DHPLC) analysis

DHPLC analysis was performed for 23 MLPA-negative patients to identify any small alterations in the *DMD* gene. All 79 exons and their flanking sequences *DMD* were separately amplified using the corresponding primers according to a previous study [24]. Unpurified PCR amplicons from these patients were mixed with those from unaffected male controls at a ratio of 1 to 1 and then denatured and cooled in a thermal cycler. DHPLC was then performed to screen DNA variations by separating heteroduplex and homoduplex DNA fragments via reverse-phase liquid chromatography using the WAVE system. The pre-treated amplicons were processed at the optimal separation gradient and temperature determined using WAVEMARKER 4.1 software. Once a PCR amplicon presented a chromatogram difference in shape or retention time from the control (wild type), the PCR products of the corresponding exon were directly sequenced to identify the specific variation; the data were then compared to the DMD database (www.dmd.nl). After that, the National Center for Biotechnology database of genetic variation (dbSNP) was queried to identify the existence of common SNPs. We used a restriction fragment length polymorphism (RFLP) analysis to rule out that a patient-associated mutation appears to be unreported SNPs.

### Short tandem repeat (STR) linkage analysis

We selected five STR markers scattering across the whole *DMD* gene and the flanking sequence. The STR primers were designed using Primer Premier 5.0 (Table S1) and synthesized by Sangon Biotech (Shanghai, China). PCR was performed followed by a denaturing polyacrylamide gel electrophoresis. Fetal gender assignment was achieved by PCR amplification of sex-determining region Y (SRY) loci (Table S1) and identification of two probes specific for the Y chromosome included in the MLPA kit. After that, we analyzed the allele type according to the electrophoresis result to confirm whether the fetus had inherited the risk allele.

### High-resolution SNP microarray assay

We used an Affymetrix Genome-wide Human SNP Array 6.0 that consists of more than 1.8 million genomic DNA markers with more than 900,000 probes each to detect copy-number variations (CNVs) and SNPs. Based on the results of the SNP array, we designed case-specific primers using Primer Premier 5.0 to amplify del/dup breakpoint junctions. Primer sequences are available on request. Long-range PCR of the patient samples and a control using TaKaRa LA Taq (TaKaRa, Dalian, China) was performed with the most likely combination of two primers according to the manufacturer's protocol. Patient-specific bands of interest were then subjected to DNA sequencing.

### Junction sequence analysis

Sequence examination was mainly based on UCSC Genome Browser (http://genome.ucsc.edu, NCBI36/hg18). We used the RepeatMasker program to evaluate interspersed repeat-element content flanking the breakpoints (100 bp upstream and 100 bp downstream), and the Blat program to determine the origin of inserted sequences at junctions. We then aligned sequences using the Nucleotide BLAST Program (http://blast.ncbi.nlm.nih.gov/) to check possible identical sequence between proximal and distal breakpoints.

## Results

### Mutation spectrum of *DMD* identified by MLPA-DHPLC-Sequencing

*DMD* deletions and duplications are major alterations of DMD patients; thus, we analyzed 119 unrelated DMD Chinese patients using MLPA and found that among them, a gross mutation was detected in 81 patients (68.1%). Of these 81 MLPA positive cases, 64 (79.0%) were *DMD* deletions, including 15 single-exon deletions and 49 multiple-exon deletions, whereas 16 (19.8%) cases were duplications, including 3 single-exon duplications and 13 multiple-exon duplications; one (1.2%) case had both deletion and duplication. However, there were four MLPA false positive cases and later, they were shown to have *DMD* point mutations (Table S2). Furthermore, 38 cases out of 59 potential female carriers who had a son with *DMD* gene rearrangement harbored a heterozygous mutation identical to the one in their affected sons.

**Figure 1:**
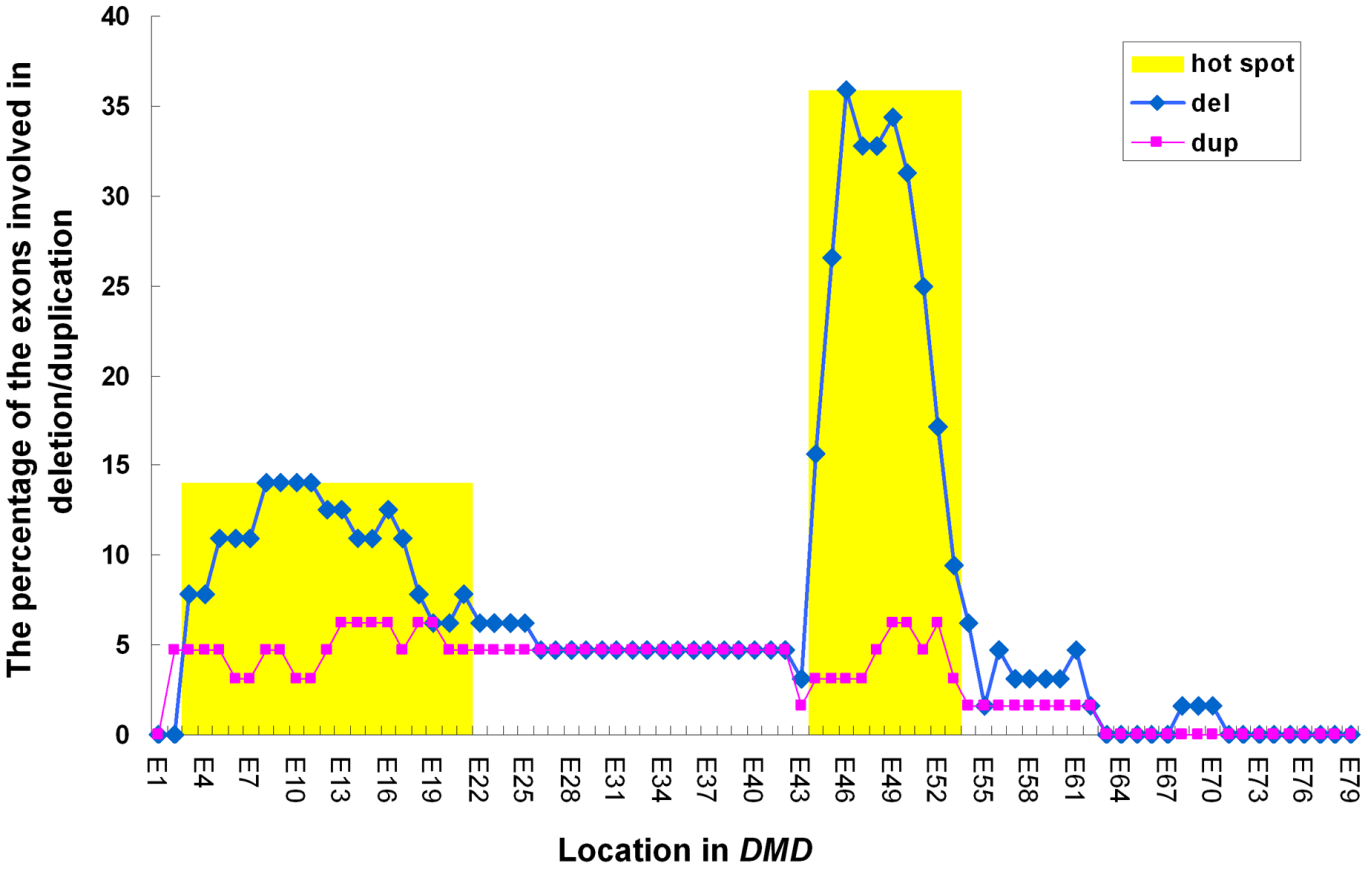
Spectrum and cumulative events of 64 DMD deletion cases and 16 DMD duplication cases. *DMD* mutations were detected using MLPA. The X-axis represents exon position, and the Y-axis represents the percentage of the exons involved in deletion (blue) or duplication (pink). Two DMD deletion hotspots, located within exon 44–53 and within exon 3–21, respectively, are shown in yellow.

**Figure 2:**
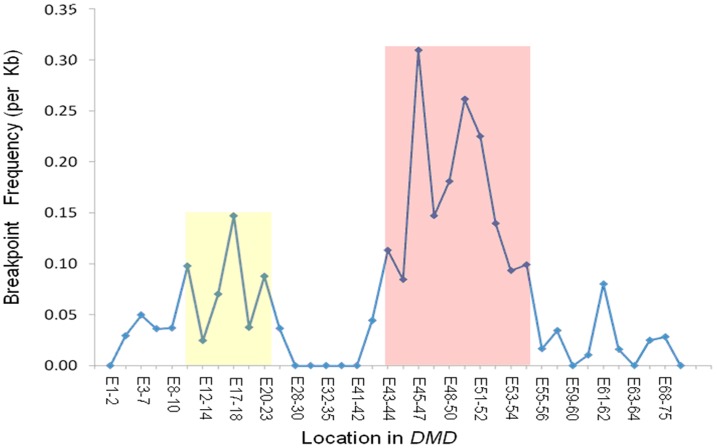
Frequency of sequence breakpoints in 64 DMD cases with exon deletions. The X-axis indicates the exon intervals for breakpoints across the *DMD* gene. The Y-axis represents the frequency of sequence breakpoints (per kb). Two DMD deletion hotspots, located within exon 43–55 and within exon 10–23, are shown in pink and yellow, respectively.

Deletion hotspots appeared to be exon 44–53 in the central region and exon 3–21 in the 5′ region of the *DMD* gene, while the distribution of duplications was not strikingly biased (Fig. 1). However, the distances between two adjacent exons are different across the *DMD* gene; therefore, the increased numbers of exon del/dup events could be caused by extended exon interval length. To confirm the potential *DMD* deletion hotspot, we re-analyzed all 64 cases with *DMD* deletions using their breakpoint frequency (per kb) (Fig. 2). We identified two deletion hotspots at exon 43–55 and exon 10–23, which has different hotspot boundaries from that of cumulative events analysis shown in Fig. 1.

In addition, we performed DHPLC analysis and then Sanger sequencing in 23 MLPA negative cases. In total, seven different *DMD* mutations were detected, five of which were novel (Table 1). In all, 85.7% (6/7) of small mutations identified in this study were nonsense mutations that resulted in a truncated non-functional dystrophin protein.

**Table 1:** Small *DMD* mutations detected using DHPLC.

Patient	Small lesions#	Protein change
**D8**	**c.9975-1G>T**	**—**
**D28**	**c.4231C>T**	p.Gln1411*
**D33**	**c.7309+5G>A**	p.Arg2401Leufs*9 &
**D35**	**c.3376A>T**	p.Arg1126*
D48	c.6352C>T	p.Gln2118*
**D56**	**c.4068_4069insGCATGAA**	p.Glu1357Alafs*2
D72	c.2281_2285delGAAAA	p.Glu761Serfs*10

& Reported in dog, previously [35].

# Novel mutations are in bold. Nucleotide sequence position is based on the annotated mRNA sequence in GenBank (accession #NM_0040006.1).

### Prenatal diagnosis using MLPA and linkage analysis

We performed a prenatal diagnosis of eight samples from the families who were at risk of giving birth to another DMD child. The proband of each family was previously screened by MLPA in our study. Among the eight samples, four had a mutation identified by MLPA; we then directly performed MLPA detection using amniotic fluid samples for the fetuses. The remaining four samples with a negative proband MLPA result, were analyzed using STR-based linkage. Out of these eight cases, five showed no clinical risk (not a carrier), one was DMD, and two had a low risk for developing DMD (Table 2).

**Table 2:** Prenatal diagnosis and clinical risk in eight cases.

Patient	Proband	Potential carrier	Fetus			
	MLPA result		Gender	MLPA result	STR	Clinical Risk
D54	44del	Neg	Female	Neg		Not carrier
D50	Neg	-	Female	-	√	Not carrier
D52	Neg	-	Female	-	√	Not carrier
D59	44del	-	Female	Neg	-	Not carrier
D66	45–46del	Neg	Female	Neg	-	Not carrier
D67	Neg	-	Male	-	√	Affected
D87	Neg	-	Male	-	√	Low risk
D103	51del	Neg	Male	Neg	-	Low risk

44del, exon 44 deleted; Neg, negative result; -, not detected; √, STR analysis.

### Genomic characteristics of *DMD* breakpoint junctions

Most deletions detected in this study were clustered in the hotspot of exons 43–55, and intriguingly, single-exon deletions most frequently affected exon 44 (Table S3). Therefore, we selected 21 DMD patients, including all of the available 14 single-exon del/dup cases and seven cases with multiple-exon deletions (residing in the major hotspot, starting from exon 45, Table S4) to determine *DMD* rearrangements at the nucleotide level.

The high-resolution SNP microarray assay enabled us to obtain the breakpoint junction sequences in all patients. There were no exactly recurrent breakpoints observed and there was also no significant difference found between single-exon del/dup and multiple-exon del/dup cases. Moreover, 1- to 5-bp microhomologies were present in 66.7% (14/21) of cases, and 2- to 34-bp small insertions were observed in 28.6% (6/21) of cases. There was only one case with neither insertions nor microhomologies (Table 3). The longest insertion was 34 bp at the junction of Sample No. 13; the 11∼30 bp of the 34-bp fragment was identical to the sequence at chromosome X 119503497-119503516 bp (Human BLAT Search program). A tiny piece of the sequence was located between the *LAMP2* (OMIM # 309060) and *CUL4B* genes (OMIM # 300304), ∼2 Mb away from the *DMD* gene. In addition, the RepeatMasker analysis showed 26 of these 42 (61.9%) junction ends overlapped with at least one kind of repeat element (Table 3), which is consistent with previous studies [25], [26] but much higher than the average frequency (35.6%) of the repetitive sequences in the *DMD* gene [26]. However, no extensive homology existed in these cases.

**Table 3:** Breakpoints and DNA sequence signatures at the junctions of 21 *DMD* deletions and duplications.

Sample i.d.	Exon del/dup	Breakpoint location^a^	Size (bp)	Microhomology or insertion^b^	RepeatMasker analysis (±100 bp)	
					Proximal breakpoint	Distal breakpoint
D54	44del	chrX: g.32,116,647_32,187,474 del	70828	TTTC	LINE: LIM4	unique
D84	44del	chrX: g.32,011,646_32,210,109 del	198464	AT insert	SINE: *Alu*Y	unique
D19	44del	chrX: g.31,969,955_32,164,747 del	194793	TA insert	LINE: L1MCa	LTR: THE1B
D59	44del	chrX: g.32,053,727_32,153,876 del	100150	CTT	unique	LTR: MSTA
D83	44del	chrX: g.31,934,564_32,161,104 del	226541	CTT	LTR: MLT1C	unique
D9	45del	chrX: g.31,859,483_31,903,740 del	44258	A	unique	unique
D16	45del	chrX: g.31,891,529_32,096,896 del	205368	—	unique	unique
D115	45del	chrX: g.31,874,683_32,009,568 del	134886	TT	unique	LTR: LTR16A; SINE: *Alu*Sp
D10	51del	chrX: g.31,690,543_31,716,601 del	26059	GT	SINE: *Alu*Sc;	LINE: L1M1
D57	51del	chrX: g.31,680,922_31,716,845 del	35924	T	unique	LINE: L1M1
D103	51del	chrX: g.31,694,717_31,708,096 del	13380	AGT	unique	unique
D106	52del	chrX: g.31,617,437_31,680,184 del	62748	AT	LINE: L1PA5	LTR: MLT1D
D77	61del	chrX: g.31,269,911_31,279,635 del	9725	TTTTTCTTGTATACAAAGAACAAATACATTACAC insert	DNA: MER112; Simple repeat: (CA)n; SINE: MIR3; DNA: MER112	SINE: *Alu*Sx
D5	2dup	chrX: g.32,938,053_33,104,879 dup	166827	CA	Simple repeat: (TATATG)n	SINE: *Alu*Sq
D66	45–46del	chrX: g.31,859,090_31,915,781 del	56692	ATGGA insert	LINE: L2; SINE: MIRb	LINE: L1MA6
D3	45–48del	chrX: g.31,770,072_31,959,729 del	189658	TTCTG	DNA: Tigger1	unique
D64	45–50del	chrX: g.31,707,077_31,997,152 del	290076	TGG	unique	LINE: L1MA4
D69	45–50del	chrX: g.31,744,481_31,994,173 del	249693	TTAAT insert	SINE: MIR3; SINE: *Alu*Sx	unique
D73	45–52del	chrX: g.31,655,640_31,909,714 del	254075	AGCT	LTR: MLT1J1	LINE: L3
D62	45–52del	chrX: g.31,650,162_31,897,770 del	247609	ATTT	Simple repeat: (TTTA)n; DNA: Looper; SINE: *Alu*Sq	unique
D23	45–52del	chrX:g.31,656,668_31,944,497 del	287830	CAAG insert	Low complexity: AT_rich	SINE: MIRm

a Coordinations of the human genome assembly (NCBI36/hg18).

b “—” indicates no microhomology or insertion.

## Discussion

To date, there is no cure for DMD. Identification of *DMD* alterations could help diagnose the disease and provide accurate genetic counseling and prenatal diagnosis for DMD patients. Moreover, detection of *DMD* alterations could also be beneficial for mutation-specific gene therapy, including exon skipping or suppression of nonsense mutations during translation [27]–[29]. In the current study, we detected *DMD* alterations in 119 unrelated DMD patients, found *DMD* mutations in 92 of those patients, and then performed prenatal diagnosis for eight families.

In the meantime, our current study identified a *DMD* mutation spectrum in Chinese DMD patients. The *DMD* mutation spectrum revealed in this study is in agreement with previous studies [5], [25]. Moreover, our current study evaluated frequency of DNA breakpoints in two ways, i.e., by counting cumulative events of exon deletions and calculating exon-deletion events of certain exon intervals. However, these two methods showed some different results (Fig. 1 and 2). We believe the breakpoint frequency (per kb) is more convictive.

In this current study, we also revealed genomic characteristics of DMD breakpoint junctions in Chinese DMD patients. We were able to determine the exact location of DNA breakpoints in all 21 cases using the SNP microarray assay. We found that all breakpoints among the 21 cases were non-recurrent, which is consistent with previous studies in other populations [12], [14]. We also found that detailed sequence signatures of breakpoint junctions were similar between single-exon del/dup cases and multiple-exon cases. According to our data, no extensive homology existed in any case; in other words, there was no evidence supporting L1-L1 or *Alu*-*Alu* recombination. The RepeatMasker analysis showed 61.9% (26/42) of cases had breakpoint junctions aligned with one or more repeat elements, which was higher than the average percentage of repeat elements in the whole *DMD* gene. Although these highly homologous sequences (such as LINEs or SINEs and especially *Alu*-elements) did not mediate these non-recurrent rearrangements, they were more likely to incite a secondary DNA structure, including stem-loop and facilitate copy-number variations. Therefore, we inferred that the repetitive elements might be involved in *DMD* gene rearrangements.

Recently, several models have been proposed to explain non-recurrent genomic rearrangements, such as the NHEJ mechanism and DNA replication-based mechanisms. The latter mainly included FoSTeS/MMBIR [22], [30], aberrant firing of replication origins [11], and replication timing associated mechanisms [12], [23]. Particularly, NHEJ is one of the prominent mechanisms for repairing double-stranded DNA breaks by joining two DNA ends in the absence of any sequence homology and then associating in a manner that tolerates nucleotide loss or addition at the junction site. In *DMD* rearrangements, our study showed some features of the DNA breakpoint sequence that could possibly contribute to *DMD* gene rearrangements. Microhomologies of 1–5 bp were present in 66.7% (14/21) of cases, which is higher than findings from other studies [12], [31]–[33]; insertions of 2–34 bp were observed in 28.6% (6/21) of all cases. Only one case presented no microhomology or insertion. The above observations are typical characteristics of an NHEJ event [34].

**Figure 3:**
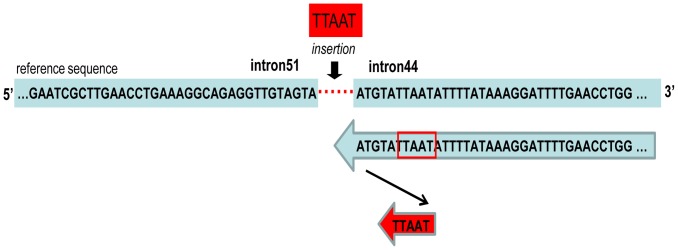
Template slippage events of Sample 18. The inserted sequence is shown in red. The red dotted line indicates the deletion region between intron 44 and intron 51. Short segments of the inserted sequence aligned with the adjacent sequence of the breakpoint. The schematic diagram shows one slippage event of the replication machinery along the template DNA.

FoSTeS/MMBIR mainly accounts for complex rearrangements. Thirty-four deletion cases and one duplication case in the current study were all simple rearrangements without any typical FoSTeS features, however, rearrangement can occur via only one FoSTeS event for each case. Most recently, it was reported that aberrant firing of replication origins underlies intragenic non-recurrent rearrangements of the *DMD* gene [11]. This model showed how various sizes of insertions were formed, an attempt to rescue the unreplicated template due to failure of replication origin [11]. In our current study, five out of six insertions (except Sample #13) could be explained by this aberrant firing of the replication origins model (Fig. 3 shows Sample #18 as an example). Hence, our current data strongly supported the findings of Ankala *et al.* [11]. As for the 34-bp insertion in Sample #13, we inferred that it most likely resulted from a FoSTeS event that template switched to a linearly far but spatially close region of the X chromosome.

The sample size for this study might not have been large enough to fully elucidate the mechanism(s) of *DMD* mutation. Our current study did comprehensively screen *DMD* mutations and reveal the frequency of *DMD* mutation breakpoints. For the first time, we characterized *DMD* breakpoints at the nucleotide level in a cohort of Chinese patients and provided insightful information for the mechanism of *DMD* rearrangements. Further study with a large sample will elucidate *DMD* mutational mechanism(s) in Chinese patients. In the future, such information will help clinicians provide accurate and reliable genetic counseling, prenatal diagnoses, and gene therapy for those at risk of DMD.

## Supporting Information

Table S1 **Primer sequences and conditions for STR analysis.**
(DOCX)

Table S2 **Small** ***DMD*** **mutations detected by MLPA.**
(DOCX)

Table S3 **Single-exon deletions cases for determining breakpoints.**
(DOCX)

Table S4 **Multiple-exon deletions cases for determining breakpoints.**
(DOCX)
