# Supplementary material for: Screening of Duchenne Muscular Dystrophy (DMD) Mutations and Investigating Its Mutational Mechanism in Chinese Patients
Source: PLoS One. 2014 Sep 22;9(9):e108038. doi: 10.1371/journal.pone.0108038 (PMC4171529; doi:10.1371/journal.pone.0108038)
Supplement: Table S1 — Primer sequences and conditions for STR analysis. (DOCX) [file pone.0108038.s001.docx]

**Table S1. Primer sequences and conditions for STR analysis**

| **Primer name** | **Sequence 5’-3’** | **Annealing** |
| --- | --- | --- |
| DMD-M1F | CACTCCTTTGTATCACTGCCAT | 54℃ |
| DMD-M1R | CCGGTTATCCGAACACTTT |  |
| DMD-M2F | ATGGGCTCAAACGATTCTCC | 60℃ |
| DMD-M2R | ACTGGGGGGCTGTGGTGTAA |  |
| DMD-M3F | AGGTCGGAGTGCAGTGGT | 60℃ |
| DMD-M3R | TGAGCCCAGAAGTTTGAGT |  |
| DMD-M4F | GAGCATTCCCAATCCACTT | 60℃ |
| DMD-M4R | TGTAACCCCAGCTACTCAG |  |
| DMD-M5F | TTCACCATGTACTCTTGCCTCT | 54℃ |
| DMD-M5R | CTTGGAAAAGTGATTTGAAAAC |  |
| DMD-M6F | TTTTCTTCCTCCTCCTCCTC | 60℃ |
| DMD-M6R | AAGCTCTAATCCTCAATGGC |  |
| DMD-M7F | GCAACTAACATATTTATTCATCC | 50℃ |
| DMD-M7R | CATGTATTCCAGAACTTAAAGC |  |
| SRY-F | GTTGTCCAGTTGCACTTCGCTGCA | 60℃ |
| SRY-R | CAGTGTGAAACGGGAGAAAACAGT |  |
