# Supplementary material for: Screening of Duchenne Muscular Dystrophy (DMD) Mutations and Investigating Its Mutational Mechanism in Chinese Patients
Source: PLoS One. 2014 Sep 22;9(9):e108038. doi: 10.1371/journal.pone.0108038 (PMC4171529; doi:10.1371/journal.pone.0108038)
Supplement: Table S2 — Small DMD mutations detected by MLPA. (DOCX) [file pone.0108038.s002.docx]

**Table S2. Small DMD mutations detected by MLPA**

| **Patient** | **Small lesions** | **Protein change** |
| --- | --- | --- |
| D3 | c.8608C>T | p.Arg2870* |
| D90 | c.2302C>T | p.Arg768* |
| D102 | c.2302C>T | p.Arg768* |
| D116 | c.5273_5286del | p.Glu1759Ilefs*9 |
