# Supplementary material for: Screening of Duchenne Muscular Dystrophy (DMD) Mutations and Investigating Its Mutational Mechanism in Chinese Patients
Source: PLoS One. 2014 Sep 22;9(9):e108038. doi: 10.1371/journal.pone.0108038 (PMC4171529; doi:10.1371/journal.pone.0108038)
Supplement: Table S3 — Single-exon deletions cases for determining breakpoints. (DOCX) [file pone.0108038.s003.docx]

**Table S3**. **Single-exon deletions cases for determining breakpoints**

| **Sample No.** | **Single-exon deleted** |
| --- | --- |
| D19 | E44del |
| D54 | E44del |
| D59 | E44del |
| D83 | E44del |
| D84 | E44del |
| D9 | E45del |
| D16 | E45del |
| D115 | E45del |
| D10 | E51del |
| D57 | E51del |
| D103 | E51del |
| D106 | E52del |
| D77 | E61del |
| D5 | E2dup |
| Total cases | 14 |
