# Supplementary material for: Screening of Duchenne Muscular Dystrophy (DMD) Mutations and Investigating Its Mutational Mechanism in Chinese Patients
Source: PLoS One. 2014 Sep 22;9(9):e108038. doi: 10.1371/journal.pone.0108038 (PMC4171529; doi:10.1371/journal.pone.0108038)
Supplement: Table S4 — Multiple-exon deletions cases for determining breakpoints. (DOCX) [file pone.0108038.s004.docx]

**Table S4. Multiple-exon deletions cases for determining breakpoints**

| **Sample No.** | **Multiple-exon deleted** |
| --- | --- |
| D23 | E45-52del |
| D62 | E45-52del |
| D73 | E45-52del |
| D64 | E45-50del |
| D69 | E45-50del |
| D66 | E45-46del |
| D3 | E45-48del |
| Total cases | 7 |
